# Carers’ Understanding of Recovery‐Oriented Practice in Mental Health Settings: A Systematic Review and Narrative Synthesis

**DOI:** 10.1111/inm.70035

**Published:** 2025-04-07

**Authors:** Birhanie Mekuriaw, Natalie Ann Cutler, Jo River

**Affiliations:** ^1^ Faculty of Health University of Technology Sydney Sydney Australia; ^2^ Department of Psychiatry, College of Health and Medical Science Dilla University Dilla Ethiopia; ^3^ Northern Sydney Local Health District Sydney Australia

**Keywords:** caregivers, mental health recovery, mental health services, qualitative research, systematic review

## Abstract

Recovery‐oriented practice is a contemporary and internationally accepted approach to mental health care. Moving away from privileging ‘clinical recovery’, it recognises and supports ‘personal recovery’, defined as living a meaningful life. Family and carers' (carers) understanding, and support of recovery‐oriented practice is crucial to the provision of comprehensive and continuous mental health care. Few studies exist on carers' knowledge and experiences of recovery‐oriented practice. We conducted a systematic review to explore carers' understanding of recovery‐oriented practice in mental health settings. A narrative synthesis was undertaken using both deductive and inductive approaches, guided by the established framework for recovery‐oriented practice by Le Boutillier and colleagues. Findings indicated that carers have a grasp of the principles and aims of recovery‐oriented care, which aligned with the selected framework. However, carers' comprehension also identified shortcomings in how recovery‐oriented practices were currently implemented in mental health services. Extending the work of Le Boutillier and colleagues, this review found that carers experienced a ‘disillusionment with mental health services’. While carers were keen to be involved and support recovery, they often felt excluded by mental health workers and the broader mental health system. This sense of exclusion led to carers feeling unsupported and disinclined to engage with the service, and this adversely affected their well‐being. Findings suggest that operationalising recovery‐oriented practice requires more genuine involvement of carers in decision‐making forums such as care planning meetings, and formal feedback mechanisms be made available to integrate their perspectives into service development.

## 1 Introduction

Recovery‐oriented practice is a contemporary approach to mental healthcare that aims to build, maintain and promote personal recovery as determined and defined by a person with a lived experience of mental health challenges (Subandi et al. 2023). While a clinical conception of recovery, which is focused on the reduction or elimination of symptoms, continues to dominate mental health service provision (Rocca and Anjum 2020), the concept of personal recovery has become central to mental health care (World Health Organization 2021), largely due to advocacy from people with lived experience of mental health challenges (lived experience) movements (Slade et al. 2012). Personal recovery has been defined as a way of living a hopeful, purposeful, meaningful and contributing life even with ongoing mental health challenges (Damsgaard and Angel 2021). The concept of personal recovery asserts the right of people with lived experience to determine their own unique goals for recovery, and to live a meaningful life with or without mental health symptoms (Commonwealth of Australia 2013). In this paper, the term ‘recovery’ refers to personal recovery.

Recovery‐oriented practice in mental health settings (e.g., community and inpatient) refers to practices that support and promote recovery (Belayneh et al. 2024; Kidd et al. 2015; World Health Organization 2021). Recovery has been promoted in mental health services by attending to the unique needs and identity of a person and fostering connection, social inclusion, hope, self‐determination and empowerment (Cutler et al. 2021; World Health Organization 2021). Such practices are transformative and vitally important beyond symptom management as they challenge stigma and discrimination as well as promote well‐being for both carers and the person they care for (Fawor et al. 2023). Although predominantly practised in high‐income countries, in low‐income countries, where mental health resources are often scarce, recovery‐oriented practices can be adapted to leverage community‐based care, informal support networks and culturally relevant interventions (Belayneh et al. 2019; World Health Organization 2021).

Optimal implementation of recovery‐oriented practice includes the involvement of carers, who play a crucial role in supporting people with lived experience in their progress towards recovery (Belayneh et al. 2025; Vera San Juan et al. 2021). In this paper, ‘carers’ refers to family members, friends or other support people (e.g., neighbours and colleagues) who provide unpaid care for a person with lived experience.

Research indicates that carers may have a limited understanding of what recovery‐oriented practice means in the context of mental health services, and how they can best support this approach (Hungerford and Richardson 2013). Despite the importance of carers in supporting recovery, little is known about carers' understanding, experience, or perceptions of recovery‐oriented practice, and to date, no systematic reviews providing a comprehensive review of the available evidence have been conducted on this topic. A more detailed understanding of how carers perceive and experience recovery‐oriented practice in mental health settings is required and would be the first step towards identifying how carers can be optimally supported to contribute and provide recovery‐oriented care. This paper seeks to address this gap by reporting on a systematic review and narrative synthesis that explored carers' understanding of recovery‐oriented practice in mental healthcare settings.

## 2 Methods

This systematic review and narrative synthesis aimed to explore carers' understanding of recovery‐oriented practice in mental health settings, including carers' perspectives, views, knowledge, attitudes, conceptualisations and experiences of recovery‐oriented practice. The review question was, ‘How do carers understand recovery‐oriented practices in mental health settings?’ The review was performed in line with Preferred Reporting Items for Systematic Review and Meta‐Analysis (PRISMA) guidelines (Page et al. 2021). The protocol was registered (PROSPERO 2023, CRD42023450196).

### 2.1 Databases and Search Strategy

Recovery‐oriented practice is a nebulous concept and is often difficult to define (Le Boutillier et al. 2015). Therefore, to determine searchable terms, an initial search was conducted in Google Scholar using the search terms: ‘carers’, ‘mental health’ and ‘recovery‐oriented practice’. The first 100 papers were reviewed to identify eligible research studies. Four studies of carers' perspectives on recovery‐oriented practice were selected as ‘marker’ papers, which allowed us to refine our search strategy and ensure these papers were captured. Search terms were then collected from the title, abstract and keywords of relevant research articles, and the final search strategy included related medical subject headings (MeSH) terms.

A systematic search of six scientific databases was conducted across Ovid/MEDLINE, EMBASE, CINAHL, PsycINFO, SCOPUS and Web of Science. The keywords combined four concepts: (carers [providing unpaid care for people with lived experience of mental health challenges]) AND (mental health [without diagnostic criteria specification] OR mental health services) AND (Recovery [with truncation to identify recovery‐oriented practice‐related phrases or terms]) AND (understanding [understanding, perception, knowledge, attitude, awareness, experience]; Data S1). Available studies were searched until 2 June 2023. Reference lists of included articles were also searched manually to identify relevant research articles.

### 2.2 Study Selection and Eligibility Criteria

Search results were imported into Covidence software to screen search results (Covidence 2023). Duplicates were removed initially, and one author (BM) conducted the title and abstract screening with regular guidance and oversight from both co‐authors. Eligible studies included primary research papers that used quantitative, qualitative or mixed‐method study designs and reported on carers' understanding of recovery‐oriented practice in mental health settings. To keep our search as broad as possible, we did not restrict inclusion based on publication year, geographical location or country income level. Studies were included if they were focused on general community and inpatient mental health settings. Papers that reported on carers with other participants such as mental healthcare workers, and people with lived experience, were only included if carers' responses could be distinguished and extracted within the broader findings. Highly specialised services, such as substance use services, eating disorder clinics, forensic mental health, child and adolescent services, and consumer/peer‐led services were also excluded as they may have different practice approaches and recovery pathways.

**FIGURE 1:**
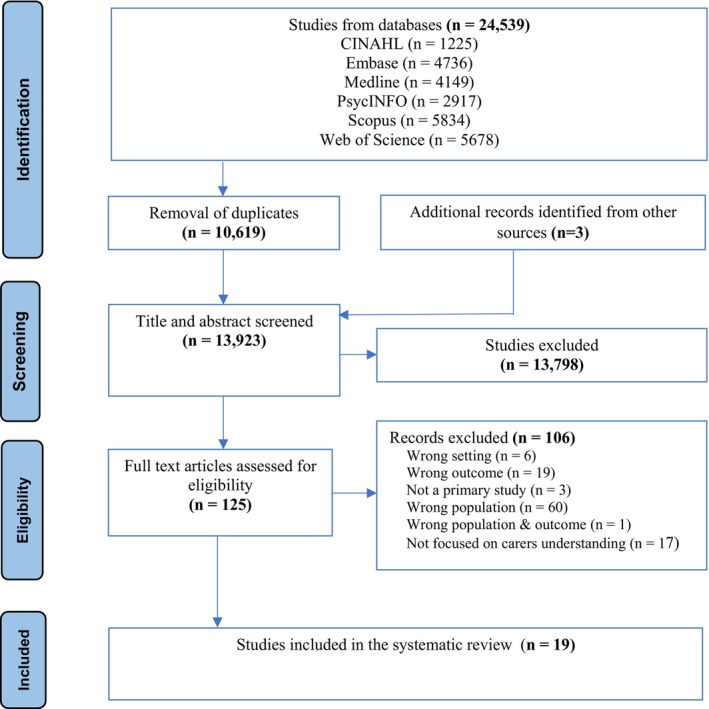
PRISMA flow diagram.

Titles identified in the electronic search were read to identify those with possible relevance. Abstracts from relevant publications were reviewed, and where they appeared to meet the inclusion criteria, the full‐text publication was obtained and assessed for eligibility. Information was shared with all authors about the decision to include or exclude studies during title and abstract screening. Papers not in English were excluded during title and abstract screening. Papers that could not be retrieved via databases or following a request to the lead author, were excluded. All full‐text papers were independently rated by two authors (BM and NC) for inclusion. Conflicts were resolved through discussion and frequent consultation with the third author (JR) to ensure decisions were more objective and reliable. The PRISMA flow diagram showing the selection process of included articles is outlined below (Figure 1).

### 2.3 Quality Assessment and Data Extraction

Since this review included qualitative, quantitative, and mixed method studies, we used the Mixed Method Appraisal Tool (MMAT; Hong et al. 2018) to evaluate the quality of each article. The appraisal tool includes two screening questions for all study types (related to research questions) and five criteria for specific methodological assessment (e.g., for qualitative, quantitative and mixed‐methods studies). The assessment questions have three possible responses: ‘Yes’, ‘No’ and ‘Can't tell’. Research articles with ‘Yes’ responses for all questions scored 100%, and a score of 60% and above was deemed high quality. Data were extracted using a data extraction proforma, which included author, year, country, aims, study design, analysis, service type, and carers' understanding of recovery‐oriented practice.

### 2.4 Analysis

The data were analysed using the narrative synthesis approach guidelines (Popay 2006), which include the following stages: (1) Selecting a framework; (2) Producing a preliminary synthesis; (3) Exploring data relationships within and between the studies; and (4) Assessing the overall robustness of the narrative synthesis. Our preliminary synthesis used a ‘convergent and integrated’ approach, which involves combining qualitative and quantitative data by ‘qualitising’ quantitative data (Stern et al. 2020).

1. *Framework selection*: This study uses a ‘best fit’ framework synthesis approach (Langlois et al. 2018). This is an established approach to exploring extracted review data using a deductive and inductive approach. We used this approach as carers' experiences might not fit with the predefined framework while detailing aspects of recovery‐oriented practice in the included studies. First, Le Boutillier et al.'s (2011) framework was used as a priori structure for the systematic review, data extraction and analysis and findings interpretation. This is an empirically derived conceptual framework that was developed from a comprehensive review of 30 international papers on recovery‐oriented practice (guidelines and research papers; Le Boutillier et al. 2011). The framework has been widely adopted in mental health services as it is highly relevant in aiding to change recovery‐oriented practice rhetoric/guidelines into practice. Le Boutillier et al.'s (2011) framework comprises four practice domains and 16 themes of mental health recovery‐oriented practice. The framework offers a comprehensive, evidence‐based set of pre‐defined constituents of recovery‐oriented practices that capture the complexity of the recovery journey. The framework integrates multiple dimensions of recovery, addressing not just clinical symptoms but also personal, social and environmental factors. Second, we explored categories and themes that emerged from the data that were not pre‐determined by Le Boutillier et al.'s framework. This involved undertaking a thematic analysis, independently coding and categorising data, and determining any additional themes that did not fit the predefined framework.
2. *Producing a preliminary synthesis*: Tabulation of extracted data was undertaken to produce a preliminary synthesis. The data were then deductively coded line by line using the predefined practice domains, themes and subthemes outlined in the framework (Le Boutillier et al. 2011). Categories and subcategories were mapped using NVivo Lumivero qualitative analysis software (Lumivero 2018). Data were also reviewed through an inductive approach to examine potential codes and themes beyond those in the chosen framework. One major theme, ‘Disillusionment with mental health services’ was identified through this approach. Terms used to code the inductive data were thematised to represent carers' experience of disillusionment and important extracts from included articles were collated and grouped.
3. *Assessment of data relationships*: Themes and domains of findings were counted to evaluate the pattern and relationship within the study and the difference in terms of characteristics (e.g., country and carers' involvement in the research design) between the studies.
4. *Assessing synthesised data robustness*: This was performed to evaluate the strength of the synthesis and determine the reliability of the evidence for drawing conclusions and extending its findings to a broader context. A quality appraisal tool (MMAT‐2018) was used to rate the quality of included papers to indicate the validity of the study conclusion. Research studies that were rated as high quality were used for initial analysis to produce preliminary synthesis. Data analysis was carried out twice, first with the high‐quality studies (*N* = 5) and second by including low‐quality studies (*N* = 2) to determine whether new categories would emerge. Open coding from the tabulated data was performed by two authors (BM and NC) first and refined with the consultation of the third author (JR).

## 3 Results

### 3.1 Study Characteristics

A total of 19 studies were included, comprising 16 qualitative studies, one quantitative study and two mixed‐method studies (Table 1). The studies were conducted between 1989 and 2020. All of the included studies were from high‐income countries including Australia (*N* = 8), United States (*N* = 3), Sweden (*N* = 3), Canada (*N* = 1), Israel (*N* = 1), United Kingdom (*N* = 1), Ireland (*N* = 1) and Singapore (*N* = 1). The studies used interviews (*N* = 8), focus groups (*N* = 6), both interviews and focus groups (*N* = 2), surveys (*N* = 2), and pretest–posttest evaluation with the self‐report survey (*N* = 1). Of the included studies, 14 (75%) included carers as well as people with lived experience and other stakeholders (e.g., mental healthcare workers). The remaining five studies (25%) included only carer participants. Approximately 335 carers were involved across the included studies. The exact number of carers is unknown because one study did not specify the number of carer participants. Only one study reported that carers were involved in the research design.

**TABLE 1:** Characteristics of included studies.

Authors and year of publication	Country of study	Study aim	Sample size	Study design	Data collection and analysis methods	Quality (MMAT)
Hungerford and Richardson (2013)	Australia	Evaluating the experience/perception of carers regarding the implementation of recovery‐oriented practice. Carers were involved in the research design and development	*N* = 10/Carers	Descriptive single case embedded study	Focus group/Interpretive phenomenological analysis	High*****
Kidd et al. (2015)	Australia	Exploring recovery and recovery‐oriented practice	*N* = 1 carer, 6 consumers, 4 clinicians	Descriptive/exploratory qualitative	Focus group/Thematic analysis	High****
McKenna et al. (2014)	Australia	Examining the current service delivery (is it operating following recovery‐oriented practice or not?)	*N* = 5 carers, 15 consumers, and staff	Narrated qualitatively/specific design not mentioned	Both in‐depth interviews and focus groups/content analysis	Low**
Martin et al. (2019)	Australia	Examine the mental health service experience of carers for the LGBTIQ community with mental health challenge	*N* = 18 carers	Exploratory qualitative	In‐depth interviews/thematic analysis	High****
Fletcher et al. (2019)	Australia	Prioritising the treatment recommendations for consumers in locked wards to make it less restrictive	*N* = 9 carers, 9 consumers, 17 staff	Participatory forum	Focus group discussion/thematic analysis	Low**
McKenna et al. (2016)	Australia	Assessing mental health service/recovery‐oriented practice	*N* = 3 carers, 11 staff, 7 consumers	Exploratory qualitative	In‐depth interview thematic analysis with an inductive approach	Low**
Hyde (2013)	Australia	Examining carers' perception of Mutual Aid Support in their caring practice	*N* = 51 carers	Mixed‐method (pre‐ and post‐training evaluation)	Pre and post‐carers narration regarding their perception of caring experience.	Low*
Wyder et al. (2018)	Australia	To examine the carers' experience of involuntary mental health admission in the context of recovery‐oriented care	*N* = 19 carers	Explanatory qualitative study	In‐depth interview/inductive thematic analysis	High****
Jormfeldt et al. (2014)	Sweden	Examining the next of kin's description of the rehabilitation process and outcome	*N* = 10 carers	Exploratory qualitative	In‐depth interview/content analysis	High***
Schröder et al. (2007)	Sweden	Examining carers' perception of psychiatric quality of care	*N* = 12 carers	Descriptive qualitative/Phenomenological approach	In‐depth interview/phenomenological analysis	High***
Engqvist and Nilsson (2014)	Sweden	Evaluating the next of kin's experience of the recovery process	*N* = 6 carers, 7 consumers	Exploratory qualitative	Semi‐structured interview	High****
Scheyett et al. (2006)	USA	Examining the views of consumers and family members on evidence‐based practices	*N* = 31 individuals/consumers and carers	Exploratory qualitative	Focus group discussion/thematic analysis	High*****
Grusky et al. (1989)	USA	Evaluate the relative importance of mental health service components as per stakeholder's perception	*N* = 77 carers, 49 staff, 82 consumers, 156 directors	Survey/cross‐sectional	Face‐to‐face interview/survey (described qualitatively)	High***
Burns‐Lynch et al. (2014)	USA	Assessing the stakeholder's experience with mental health crisis service	*N* = 33 carers, 20 consumers, 73 staff	Mixed method study	Quantitative descriptive and qualitative narration	Low**
Wong et al. (2019)	Singapore	Examining the perspectives of consumers and caregivers on case management	*N* = 19 carers, 47 consumers	Exploratory qualitative	Focus group discussion/thematic analysis	High*****
Galimidi and Shamai (2022)	Israel	To evaluate the recovery process of parents and consumers	*N* = 30 carers, 15 consumers	Grounded theory	In‐depth interview/Individual, dyadic, and triadic levels of narration	High*****
Fox et al. (2015)	UK	To assess the carer's understanding of recovery and their perceived relationship	*N* = 14 carers	Exploratory qualitative	Focus group discussion/thematic analysis	Low*
Grady et al. (2014)	Ireland	Describing the perception and experience of service users and carers in acute mental health setting	*N* = 6 carers, 13 service users	Exploratory qualitative	In‐depth interview/thematic analysis	High***
Pope et al. (2019)	Canada	Examining the views of stakeholder's responsibility for care	*N* = 12 carers, 13 service users, 18 treatment providers	Descriptive qualitative	Both focus group discussion and interview/thematic analysis	High***

*Note:* * = indicates study quality strength.

Abbreviation: *N*, number.

### 3.2 Carers' Understanding of Recovery‐Oriented Practice

The analysis of included articles, guided by a priori framework (Le Boutillier et al. 2011), revealed several key themes. The prevailing domains and themes aligned with Le Boutillier et al.'s framework, including: Domain 1 Promoting citizenship—‘Stigma and seeing beyond the service user’ and ‘Social inclusion and meaningful occupation’; Domain 2 Organisational commitment—‘Quality improvement’ and ‘Care pathways’; Domain 3 Supporting personally defined recovery—‘Autonomy and individuality’, ‘Informed choice’, ‘Peer support’, ‘Strength‐based support’ and ‘Holistic approach’; and Domain 4 Working relationships—‘Partnership’ and ‘Promoting hope’. These domains and themes are presented below. An additional theme determined inductively beyond Le Boutilier et al.'s framework is also presented, ‘Carers' disillusionment with mental health services’.

#### 3.2.1 Domain 1: Promoting Citizenship

Le Boutillier et al. (2011) domain of promoting citizenship recognises the need for recovery‐oriented services to promote the humanity and rights of people with lived experience, including their right to inclusion, connection and meaningful life within and beyond the limits of mental health symptoms (Le Boutillier et al. 2011; Ponce and Rowe 2018). Two‐thirds of the included studies (*N* = 14) reported on carers' perception of whether citizenship was promoted in mental health services. Within the included studies, carers' views aligned with two themes in this domain: ‘Stigma and seeing beyond the service user’ and ‘Social inclusion and meaningful occupation’.

##### 3.2.1.1 Stigma and Seeing Beyond the ‘Service User’

This theme not only recognises a person's unique right to define recovery but also relates to social stigma and discrimination. Eight of the studies included descriptions of carers' experiences of stigma (Burns‐Lynch et al. 2014; Fox et al. 2015; Galimidi and Shamai 2022; Grady et al. 2014; Hungerford and Richardson 2013; Pope et al. 2019; Scheyett et al. 2006; Schröder et al. 2007). Some carers reported feeling stigmatised in mental health services and community and experienced shame. They noted that this shame was largely borne from the stigma and discrimination directed towards people with lived experience:

I don't think there's any group you can make fun of, except the mentally ill and maybe WASPS. (Scheyett et al. 2006, 250)

Carers noted that they expected mental healthcare workers to dispel stigma, but they found that, instead, they could reinforce a sense of shame by implying that mental health challenges were a ‘taboo’ subject. As one carer described:

Before this happened, I was foolish enough to believe that things had progressed, that it was no longer a taboo and this form of health care had also advanced. You get a psychiatric illness just as you get another illness, and mental health care should be on the same level as physical health care, but I didn't find it that way, instead, it was still all so hush‐hush. In other words, it's a taboo and disgusting to be mentally ill. (Schröder et al. 2007, 310)

In line with personal recovery, carers in some studies defined recovery beyond narrow biomedical definitions, but they also described a need to understand mental health diagnoses and to be able to model acceptance of these. Some carers perceived this as an important step in combating stigma, accepting the person they care for, and supporting that person's recovery. This is evident from the following quotes:

Father ‘Our acceptance of the illness and our acceptance of him as a person encouraged and reinforced him’.

Mother ‘I feel that I have accepted what [my son] has. The illness is clearer to me, and so I think that I am clearer now to myself and know more [about] how to help him and to convey clearer messages of support…. It is experienced by him as more appropriate and it is healthier for me’ (Galimidi and Shamai 2022, 415).

##### 3.2.1.2 Social Inclusion and Meaningful Occupation

Ten of the included studies (Engqvist and Nilsson 2014; Fox et al. 2015; Grady et al. 2014; Grusky et al. 1989; Hungerford and Richardson 2013; Hyde 2013; McKenna et al. 2014; Pope et al. 2019; Scheyett et al. 2006; Wyder et al. 2018) reported on carers' perceptions of the relevance of mental health services in promoting community reintegration for people with lived experience. This included services meeting the basic needs of people with lived experience to live in the community, including access to food, income and housing, as well as supporting social inclusion, community networking and employment opportunities (Scheyett et al. 2006). Carers perceived that their unwavering and persistent support for the person with lived experience could strengthen family bonds and enhance mutual understanding, as they faced mental health challenges together. One author noted that this could aid both carers and the person with lived experience in experiencing a sense of recovery together (Engqvist and Nilsson 2014).

Careful discharge planning of people with lived experience was also understood by carers to be important for social inclusion (Wyder et al. 2018). However, Wyder et al. (2018) found the majority of carers in their study did not receive adequate information on the discharge of the person with lived experience, and mental healthcare workers did not collaborate with carers to reintegrate people with lived experience into the community. This left carers feeling stranded:

So at the moment, there's no plan in place or anything. It's just do it yourself.

No discharge plan or anything. They didn't even explain the medications to us, how he would take it, what times to give it to him. Just said “Bye”. They gave us a bag of medication. That's weird. (Wyder et al. 2018, 326)

Five studies (Fletcher et al. 2019; Grusky et al. 1989; Pope et al. 2019; Scheyett et al. 2006; Schröder et al. 2007) described how carers understood that meaningful activities and occupations for people with lived experience were important for inclusion and promoting recovery. Carers indicated that activities not only reduced boredom for people with lived experience but also promoted a sense of purpose and meaning. For example, one carer stated:

It's just a question of having something to occupy yourself with. They [person with lived experience] can do something with their hands, for instance – at least it's a form of occupation and gives life a bit of meaning. (Schröder et al. 2007, 312)

Carers also described how a day programme was important as it promoted a sense of meaning and social engagement, including the sharing of personal experiences, which contributed to the health and well‐being of people with lived experience (Grady et al. 2014). They also recognised the importance of meaningful paid employment, as it could help individuals regain socially valued roles that may have been disrupted by acute mental health challenges (Pope et al. 2019).

#### 3.2.2 Domain 2: Organisational Commitment

The domain of organisational commitment is characterised by mental health services having a ‘recovery vision’, reflecting a health service's dedication to creating a healthcare environment that promotes recovery‐oriented practice, and prioritises people with lived experience needs over service‐centric priorities (Le Boutillier et al. 2011). Within the included studies, carers' perspectives aligned with two themes under Le Boutillier et al. (2011) organisational commitment domain: ‘Quality improvement’ and ‘Care pathway’.

##### 3.2.2.1 Quality Improvement

In one study (Kidd et al. 2015), findings indicated that carers believed lived experience and carer involvement in services were indispensable. However, they noted shortcomings in the meaningful involvement of people with lived experience and carers in the design, delivery and evaluation of mental health services. Carers particularly noted that mental health services needed to be more active in empowering people with lived experience to inform service development:

The [mental health] service would need to do work on ways in which individual consumers can have a voice and be supported to use their own knowledge and experience to inform the development of an individualised service. (Kidd et al. 2015, 42)

##### 3.2.2.2 Care Pathways

In three studies (Burns‐Lynch et al. 2014; Grady et al. 2014; Schröder et al. 2007), carers expressed the perception that mental health services need to improve care pathways for people with lived experience. Carers noted that care pathways were often complex, and delays were common. As one carer stated:

The outcome was that she was transported to the hospital … and after about 10 h there, was admitted involuntarily. We had to wait a long time for a psychiatrist to see her and then for a court order from a judge. (Burns‐Lynch et al. 2014, 121)

Carers also described a need for more flexible and accessible mental health service delivery, including 24‐h service availability and having phone contact with mental healthcare workers as needed (Grady et al. 2014).

#### 3.2.3 Domain 3: Supporting Personally Defined Recovery

This domain of supporting personally defined recovery relates to providing care that is responsive to an individual's unique recovery needs. This domain indicates that recovery‐oriented care includes collaboration, empowerment, holistic (clinical and psychosocial) and strengths‐based care, as well as opportunities to access peer support (Le Boutillier et al. 2011; Leamy et al. 2011). Fourteen of the included studies described carers' understanding of recovery‐oriented practices which were aligned with doma of ‘Autonomy and individuality’, ‘Informed choice’, ‘Peer support’, ‘Strength‐based support’ and ‘Holistic approach’.

##### 3.2.3.1 Autonomy and Individuality

In four studies (Galimidi and Shamai 2022; Jormfeldt et al. 2014; McKenna et al. 2016; Scheyett et al. 2006), carers described understanding the need to promote self‐determination and autonomy for people with lived experience as fundamental to recovery. Carers indicated they viewed the capacity of mental healthcare workers to establish therapeutic relationships and provide individualised interventions as crucial to supporting the autonomy and recovery of people with lived experience. This included the provision of opportunities for a person with lived experience to undertake self‐care:

It's vital that staff avoid helping the client too much when he has the ability to take care of himself without any help. It's important that staff are able to vary their support in relation to individual needs. (Jormfeldt et al. 2014, 5)

##### 3.2.3.2 Informed Choice

In nine of the included studies, carers reported an understanding of the need to focus on informed choice. In two studies (Grady et al. 2014; Schröder et al. 2007), carers perceived that having information regarding the progress of the person they were caring for enabled them to provide effective support, including being able to support the decision‐making of people with lived experience. Being able to communicate with mental healthcare workers and obtain information about the person they were caring for was also understood by carers as essential for alleviating carers' concerns and reducing their experience of stress (Wyder et al. 2018).

However, across nine studies (Fletcher et al. 2019; Grady et al. 2014; Hungerford and Richardson 2013; Hyde 2013; Jormfeldt et al. 2014; Scheyett et al. 2006; Schröder et al. 2007; Wong et al. 2019; Wyder et al. 2018), carers' described a lack of information sharing between mental healthcare workers, carers and people with lived experience. Some carers noted that mental healthcare workers failed to provide information about the person they cared for, which carers perceived as hindering their ability to support recovery for the person with lived experience. Indeed, carers indicated that, in mental health settings, they were often not informed about recovery‐oriented practices, and how carers might support and enhance this (Hungerford and Richardson 2013). The following quotes are indicative of the carers' experience:

[Carers] need some training, direction, support, and some knowledge … And that [isn't being] provided to us … [Carers] don't know what to do to actually be useful in supporting recovery…I can't say that I actually helped with recovery because I wasn't told what to do and couldn't imagine what I was meant to do to be effective. (Hungerford and Richardson 2013, 5)

##### 3.2.3.3 Peer Support

Two of the included studies indicated that carers, as well as people with lived experience, benefited from peer support. Carers perceived that sharing their own experiences of caring with other carers, as peers, could substantially alleviate their stress and serve as an inspiration in supporting recovery for the person they were caring for (Burns‐Lynch et al. 2014; Fletcher et al. 2019).

##### 3.2.3.4 Strength‐Based Support and Holistic Approach

Carers recognised the inherent strengths and knowledge of people with lived experience, perceiving this as crucial for recovery‐oriented practice. A strengths‐based approach was regarded by carers as not only supporting recovery but also nurturing relationships within the family and broader social circle. Recognising the inherent strengths and knowledge gained through lived experience was understood by carers as a means of empowering people with lived experience to lead their own recovery process (Galimidi and Shamai 2022; Kidd et al. 2015; Schröder et al. 2007).

Finding ways to have conversations with people about how they have responded to the experience of mental illness, what it taught to them about themselves, or what strengths and skills they have drawn on to get through it is part of the conversation to have. That is the richness which you can draw on to further support the person. They will find ways through it, they probably already have, but they may not have noticed. (Kidd et al. 2015, 42)

In eleven studies, carers noted a need for mental health services to provide psychosocial support in addition to clinical care, including emotional support for carers as well as for people with lived experience (Burns‐Lynch et al. 2014; Engqvist and Nilsson 2014; Fletcher et al. 2019; Grusky et al. 1989; Hungerford and Richardson 2013; Jormfeldt et al. 2014; Martin et al. 2019; Scheyett et al. 2006; Schröder et al. 2007; Wong et al. 2019; Wyder et al. 2018). As one carer noted, this did take place in some mental health settings:

She [staff] do check on my sisters, my another sister and my wellbeing also at the same time on the patient. (Wong et al. 2019, 601)

In contrast, carers in the Wyder et al. (2018) study perceived that mental healthcare workers were focused on clinical recovery, particularly symptom management with medication, rather than on holistic or recovery‐oriented care. Carers in the study were troubled by the lack of opportunities for people with lived experience to discuss their past experiences and emotional well‐being, including with counsellors in the mental health setting (Wyder et al. 2018).

#### 3.2.4 Domain 4: Working Relationships

This domain acknowledges the importance of active empowerment and participation of people with lived experience in the course of their recovery and realising their capabilities to influence the recovery process (Le Boutillier et al. 2011). The domain relates to mental healthcare workers having a genuine interest in working with individuals and carers to promote recovery through the mechanism of therapeutic relationships. Within the included studies, carers' perspectives aligned with two themes in this domain: ‘Partnership’ and ‘Promoting hope’.

##### 3.2.4.1 Partnership

In ten of the included studies (Grady et al. 2014; Hungerford and Richardson 2013; Jormfeldt et al. 2014; Kidd et al. 2015; Martin et al. 2019; McKenna et al. 2016; Scheyett et al. 2006; Schröder et al. 2007; Wong et al. 2019; Wyder et al. 2018), carers recognised that good working relationships between mental healthcare workers and people with lived experience promoted confidence, self‐determination, autonomy and recovery, and could prevent stigma (Scheyett et al. 2006). Carers also understood that a therapeutic relationship was essential for partnership and developing a sense of mutual respect, and could enhance the capacity of an individual to express their needs (Fox et al. 2015).

##### 3.2.4.2 Promoting Hope

Two studies reported carers' understanding of the substantial impact that fostering hope can have on recovery for people with lived experience. This included the understanding that recovery goal setting and flexible service provision in mental health settings (e.g., providing overnight leave) were important for people with lived experience to believe in their ability to recover and have hope in the recovery process. Carers also understood that providing opportunities for people with lived experience to recognise their own strengths and to re‐engage with the broader community inspired hope for both people with lived experiences and their carers (McKenna et al. 2014).

Many carers, however, perceived that mental healthcare workers often communicated a bleak outlook regarding the prognosis of people with lived experience, especially during their initial visit to mental health services. This could result in carers feeling hopeless, and therefore less motivated to support the person's recovery process (Fox et al. 2015).

### 3.3 Carers' Disillusionment With Mental Health Services

Carer disillusionment with mental health services was identified as an emergent theme through inductive analysis, as it did not align with the themes of the a priori framework of Le Boutillier et al. (2011). The theme of carers' disillusionment was noted across six of the included studies (Burns‐Lynch et al. 2014; Hungerford and Richardson 2013; Kidd et al. 2015; Martin et al. 2019; Scheyett et al. 2006; Wyder et al. 2018) and relates to carers' descriptions of the dissonance between the rhetoric of recovery‐oriented practice in mental health services and carers' reality of experiences with mental health service delivery.

Despite services claiming to support recovery, carers perceived them as failing to practise in recovery‐oriented ways and meet the recovery needs of people with lived experience and their carers, leading to an increased burden on carers. As one carer noted:

Whenever I go to the acute inpatient unit and see the word ‘RECOVERY’ in big letters it makes me feel rather ill because I think it's bitterly ironic. (Hungerford and Richardson 2013, 15)

In some cases, mental health service provision was perceived by some carers as the opposite of recovery‐oriented. These included descriptions of circumstances where communication and cooperation between mental healthcare workers, carers and people with lived experience were absent. In some cases, mental healthcare workers were perceived by carers as disrespectful, neglectful and even abusive towards people with lived experience. Carers also perceived that compulsory treatment and restrictive practices were continued beyond the period necessary, in contradiction to recovery‐oriented service rhetoric (Burns‐Lynch et al. 2014). Services could also fail to be inclusive. In one study, where carers were providing care for a gender‐diverse person, carers experienced a lack of access to services and a lack of inclusivity of gender‐diverse people and their carers. In the same study, the delivery of forced treatment was perceived by carers as unnecessary and traumatising for the person with lived experience (Martin et al. 2019). As one carer explained:

I have rarely been offered carer support services and experienced one unnecessary treatment order [for a person with lived experience] resulting in traumatising experience in hospital through forced injections. (Martin et al. 2019, 34)

Carers were left feeling uncertain about whether they would seek mental health support for the person with lived experience in the future (Martin et al. 2019).

They also indicated that the mental healthcare system was under‐resourced and unable to provide adequate recovery‐oriented practices for those who needed them. Many carers were concerned about service quality and availability, stating, ‘There is nothing for the consumers’ (Scheyett et al. 2006, 252). Some carers felt that mental healthcare workers were transferring a greater share of responsibility to carers under the guise of consumer‐led service (Hungerford and Richardson 2013). This inductive theme encapsulates carers' descriptions of divergence between the espoused principles of recovery‐oriented practice and the reality of their experiences of mental health services. In some cases, carers observed approaches to care by mental healthcare workers that were contradictory to recovery‐oriented practice. This led carers to feel an increased level of burden, and reduced sense of hope, confidence and motivation to engage with mental health services.

### 3.4 Exploring Data Relationships

Thematic vote counting was undertaken using the Le Boutillier et al. (2011) framework. The results illustrate the pattern of themes and domains that were identified across the included studies (Table 2). Of the 16 themes of recovery‐oriented practice outlined in the framework, each of the included studies in this systematic review covered an average of 3.6 themes (22.6%). Each study has 2–7 recovery‐oriented practice descriptions that align with themes in the framework. Recovery‐oriented practice themes that aligned most with the included studies were: holistic approach (11 studies), partnership (10 studies), social inclusion (10 studies) and informed choice (9 studies). Themes under the ‘Organisational commitment’ domain were less described in the included studies as compared to others. Only two themes were found to have alignment (quality improvement (1 study) and care pathway (3 studies)). The newly identified theme ‘disillusionment with mental health services’ was counted alongside the practice domains of Le Boutillier et al. (2011) framework and reported across 6 included studies (Table 3). The pattern of domains between studies, based on their characteristics (country and carer involvement in the research design), was also examined. This indicated that the domain ‘Supporting personally defined recovery’ was most strongly reflected by carers in the majority of included studies (16 studies or 84%). Included studies were distributed across practice domains with no significant difference in country. Of the included studies, carers were involved in the study design and development of only one study.

**TABLE 2:** Thematic vote counting using a predefined recovery‐oriented practice framework.

Authors and Publication year	Promoting citizenship				Organisational commitment					Supporting personally defined recovery					Work relationship		Total
	Beyond service users	Users right	Social inclusions	Meaningful occupation	Recover y vision	Workplace support structure	Quality improvement	Care pathway	Workforce planning	Individuality	Informed choice	Peer support	Strength focus	Holistic approach	Partnership	Instilling hope	
Hungerford and Richardson (2013)	✔	✔	✔								✔			✔	✔		6
Kidd et al. (2015)							✔						✔		✔		3
McKenna et al. (2014)			✔													✔	2
Jormfeldt et al. (2014)										✔	✔			✔	✔		4
Hyde (2013)			✔								✔						2
Engqvist and Nilsson (2014)			✔											✔			2
Galimidi and Shamai (2022)	✔									✔			✔			✔	4
Fox et al. (2015)	✔		✔														2
McKenna et al. (2016)										✔					✔		2
Fletcher et al. (2019)				✔							✔	✔		✔			4
Scheyett et al. (2006)	✔		✔	✔						✔	✔			✔	✔		7
Schröder et al. (2007)	✔			✔				✔			✔		✔	✔	✔		7
Wong et al. (2019)											✔			✔	✔		3
Grusky et al. (1989)			✔	✔										✔			3
Burns‐Lynch et al. (2014)	✔							✔				✔		✔			4
Grady et al. (2014)	✔		✔					✔			✔				✔		5
Pope et al. (2019)	✔		✔	✔													3
Martin et al. (2019)														✔	✔		2
Wyder et al. (2018)			✔								✔			✔	✔		4
Total	8	1	10	5	0	0	1	3	0	4	9	2	3	11	10	2	69

**TABLE 3:** Vote counting of domains outlined in the predefined framework and newly identified theme from the included studies.

Study ID	Country	Carers involvement in the research design	Promoting citizenship	Organisational commitment	Supporting personal recovery	Stakeholder's working relationship	Disillusionment
Hungerford and Richardson (2013)	Australia	Yes	✔		✔	✔	✔
Kidd et al. (2015)	Australia	None		✔	✔	✔	✔
McKenna et al. (2014)	Australia	None	✔			✔	
Martin et al. (2019)	Australia	None			✔	✔	✔
Fletcher et al. (2019)	Australia	None	✔		✔		
McKenna et al. (2016)	Australia	None			✔	✔	
Hyde (2013)	Australia	None	✔		✔		
Wyder et al. (2018)	Australia	None	✔		✔	✔	✔
Jormfeldt et al. (2014)	Sweden	None			✔	✔	
Schröder et al. (2007)	Sweden	None	✔	✔	✔	✔	
Engqvist and Nilsson (2014)	Sweden	None	✔		✔		
Scheyett et al. (2006)	USA	None	✔		✔	✔	✔
Grusky et al. (1989)	USA	None	✔		✔		
Burns‐Lynch et al. 2014	USA	None	✔	✔	✔		✔
Wong et al. (2019)	Singapore	None			✔	✔	
Galimidi and Shamai (2022)	Israel	None	✔		✔	✔	
Fox et al. (2015)	UK	None	✔				
Grady et al. (2014)	Ireland	None	✔	✔	✔	✔	
Pope et al. (2019)	Canada	None	✔				
Total			14	4	16	12	6

## 4 Discussion

This systematic review examined carers' understanding of recovery‐oriented practice in mental health settings. The findings illustrated carers' perspectives and experiences across 19 primary research studies published in the past three and a half decades. It was evident from the review that carers have an understanding of the principles and aims of recovery‐oriented practices, as resonant with Le Boutillier et al. (2011) framework Domains 1–4, including Promoting citizenship, Organisational commitment, Supporting personally defined recover and Working relationships. An additional theme determined inductively beyond Le Boutilier et al.'s framework was also found and examined, carers' disillusionment with mental health services.

Taken together, findings highlight the challenges that carers face in caring for someone with lived experience and interacting with mental health services. Carers indicated a need for inclusive and recovery‐oriented practice in mental health settings, including the need to be responsive to unique recovery needs, to recognise strengths, foster hope, combat stigma and shame about mental health, and support social inclusion, meaningful occupation and self‐determination of people with lived experience.

The findings also indicate that carers viewed their efforts to support the person they cared for to resume socially valued roles in the community as essential for the collective recovery of both carers and the person they cared for, highlighting the interconnected nature of recovery and its relevance to carers as well as people with lived experience. This finding aligns with the concept of ‘relational recovery’, which views recovery through the lens of human interdependence, and recognises the potential for carers to have their own recovery needs due to the challenges of supporting the recovery of another person (Price‐Robertson et al. 2017; Wyder et al. 2022). Genuine integration of carer support into treatment plans, providing training for professionals on relational recovery practices and developing measurable strategies are crucial steps to assess and address both carers and people with lived experience needs.

Our study indicates that carers perceived mental healthcare workers as having an important role in including carers and people with lived experience in recovery‐oriented care. This includes a responsibility to provide timely information to both carers and persons with lived experience, and to include them both in decision‐making and planning. Systematic reviews have previously reported the importance of healthcare workers' engagement with carers in recovery‐oriented services, including the value of timely information and empowering carers (Ekberg et al. 2021; Kim et al. 2024), and carers' engagement in decision‐making and planning (Petkari et al. 2021). Carers also indicated a need for mental healthcare workers to support them and alleviate stress related to their caregiving role. This indicates that targeted support for carers is essential to their well‐being and addresses the significant stress associated with their caregiving role.

Despite carers demonstrating an understanding of the principles of recovery‐oriented practice in mental health settings, this systematic review indicates that carers consistently experienced barriers to engaging with mental healthcare workers to enable them to support and promote recovery‐oriented care. This was evident when carers reported that services could fail to support the personally defined recovery of the person with lived experience and continue to focus on symptom reduction by focusing heavily on medication (Wyder et al. 2018). This carers' account highlights the common tension in mental health settings where services prioritise symptom reduction/medical approach over supporting personally defined recovery (Ørjasæter and Almvik 2022). It was also evident in the lack of information shared with carers, as well as the lack of involvement of carers in decision‐making and planning, which impacted carers' ability to support the person they care for (Hungerford and Richardson 2013). These findings indicate that mental health services can not only fail to promote recovery for the person with lived experience but also that a lack of engagement with carers can impact carers' capacity to support recovery. These issues point to systemic challenges in mental health services, which often fail to embrace collaborative care involving carers fully, a point that has been highlighted in another study (Waldemar et al. 2016).

Our review found that carers also frequently observed and experienced inconsistencies between intended policy directions and organisational commitments to recovery‐oriented practices in mental health services. For example, carers noted that they were excluded from care and discharge planning, which undermined both their caregiving role and the continuity of care. A recent study confirms that individuals with lived experience are often discharged with insufficient carer involvement (Shimange and Shilubane 2023). The involvement of carers in discharge planning is not only vital for a successful transition from inpatient services to the community (Petkari et al. 2021), but it can also lower the financial costs associated with care for mental health services (Susanti et al. 2018). While mental health policies and plans espouse the involvement of carers as an essential component of recovery‐oriented practice (Commonwealth of Australia 2013), these findings indicate there continues to be a lack of meaningful engagement of carers in mental health services. This suggests that translating recovery‐oriented principles into practice remains challenging within mental health services. Despite the rhetoric of recovery‐oriented care, clinical recovery discourse continues to dominate, with a continued emphasis on medication treatment.

The lack of involvement of carers in mental health service provision may not only be a practical shortcoming of mental health services but also a critique of widely used recovery frameworks such as ‘CHIME’, which do not address the carers' experience (Poon et al. 2024; Wyder et al. 2022). Our findings show that carers viewed their exclusion from care as a breach of the principles of recovery‐oriented practice, which left them feeling neglected, frustrated, unsupported and invisible. Feelings of invisibility and being neglected can have a substantial impact on carers' well‐being (Susanti et al. 2018). The exclusion of carers may reflect cultural norms within health services, which have been largely shaped by Western conceptions and medico‐legal frameworks that emphasise individual treatment and personal recovery. While such frameworks acknowledge carers, they often overlook the interconnected and collective dynamic of care and recovery within the context of families and communities (Panadevo et al. 2024). Abou Seif et al. (2022), has referred to carers as ‘invisible experts’ in mental health settings, and noted that the exclusion of carers from recovery‐oriented practice had negative psychological and physical impacts on carers (Abou Seif et al. 2022), highlighting the need for a more inclusive recovery model.

In addition to the burden resulting from carers' lack of inclusion, this review's findings show that carers may internalise the pervasive social stigma associated with caring for a person with lived experience. This phenomenon has been described as ‘affiliated stigma’ and is associated with increasing carer burden and reluctance to engage with mental healthcare services (Kaggwa et al. 2023). Instead of challenging stigma, our findings suggest that, at times, mental healthcare workers may perpetuate it. Likewise, rather than conveying hope, which is a key attribute of recovery‐oriented practice (Commonwealth of Australia 2013), the findings indicate that mental healthcare workers may sometimes convey a bleak outlook regarding the prognosis of the person with lived experience. These practices not only impacted people with lived experience but also left carers feeling hopeless and marginalised, with few avenues for support. The compounding of stigma and the quelling of hope run counter to recovery‐oriented approaches (Jahn et al. 2020).

An important finding in this systematic review was the disillusionment experienced by carers due to the gap between their expectations for recovery‐oriented care and their actual experiences in mental health services. Despite the rhetoric of recovery, carers noted a limited application of recovery‐oriented care in mental health settings. Additionally, carers reported that, at times, services undermined recovery due to unnecessary use of restrictive practices and forced treatments, which could be experienced as traumatising to carers as well as those they care for. These factors eroded carers' trust in the mental health system and could lead to a reluctance to engage. Another study has highlighted carers' experience of becoming disillusioned due to the quality of mental health services (Speirs et al. 2023). Building on this, our findings suggest that, for carers, disillusionment can be specifically related to the gap between the rhetoric of recovery‐oriented practice and the reality of practices that are exclusionary, coercive and traumatising.

## 5 Limitations

While our study is the first that we know of to synthesise findings on carers' understanding and experiences of recovery‐oriented practice in mental health services, it has several limitations. The included studies were in English and based in high‐income countries with one study conducted in a non‐Anglo/American/European cultural setting. As recovery is an inherently culturally bound concept, the finding may not reflect the carers' perception of recovery‐oriented practice in low and middle‐income countries or non‐Western contexts. Additionally, we did not include specialised mental health services (e.g., substance use services, eating disorder clinics, forensic mental health, child and adolescent services, and consumer/peer‐led services), which may limit the applicability of the findings to these contexts.

Many of the included studies scored low in quality appraisal, and carers' perspectives were often reported in conjunction with those of mental healthcare workers or people with lived experience. Additionally, evidence regarding carers' perspectives on some of Le Boutillier et al.'s (2011) domains of recovery‐oriented practice were lacking in the included studies, with some categories only represented by one or two studies. This may signify the need for more high‐quality accounts of carers' views or experiences, particularly those focused exclusively on carers' perspectives of recovery‐oriented practice. Further research is required to explore the perspectives of carers and others, such as mental healthcare workers and individuals with lived experience, on the factors that impede or facilitate carer engagement in recovery‐oriented practice.

## 6 Conclusion

The findings of our study showed that carers understood the elements of recovery‐oriented practice as outlined in the practice domains in Le Boutillier et al.'s (2011) framework. However, carers perceived a significant disconnect between the rhetoric and reality of recovery‐oriented practice within mental health services. The findings indicate that carers' experience of services privileged clinical recovery over personal recovery. The result of this was a devaluing of, and failure to include, carers in decision‐making, planning and service development, and at times, a perpetuation of stigma and shame. For carers, these experiences left them feeling invisible and unsupported, and undermined their trust in and engagement with mental health services. Carers also perceived these experiences as impacting the recovery of the person they cared for. This study's findings showed a need for a greater understanding of the factors that promote or inhibit engagement between carers and mental healthcare workers, as a means to enhance genuine recovery‐oriented support for carers and people with lived experience welfare.

## 7 Relevance for Clinical Practice

This systematic review demonstrates that translating recovery‐oriented principles into practice, particularly in relation to carer empowerment and engagement, remains a challenge in mental health settings. Addressing systemic barriers to carer engagement may require multifaceted approaches, including ensuring that mental healthcare workers are well versed in the concepts of recovery‐oriented care and carer engagement, and ensuring resources and leadership are genuinely committed to these practices. Carer engagement requires enhanced communication, provision of information and involvement of carers in service design, service delivery and evaluation, based on explicit domains, such as those outlined by Le Boutillier et al. (2011). Developing tailored frameworks that enhance carers' capacity for effective engagement in mental health settings is crucial to alleviating carers' burden and promoting well‐being, as well as improving recovery outcomes not just for people with lived experience but also for their carers.

## Author Contributions

All authors participated in conceiving the systematic review idea and developing its protocol. B.M. searched selected databases with frequent consultation from J.R. and N.C. All authors participated in screening the retrieved papers independently using predefined inclusion criteria (B.M. was responsible for screening all the papers while J.R. and N.C. shared an equal number of papers to screen). J.R. was involved in resolving conflicts that occurred during screening. B.M. conducted data extraction with frequent assistance from both authors. B.M. and N.C. were responsible for analysing the data and producing a preliminary synthesis with the consultation of J.R. B.M. composed the first draft of the manuscript, and several thorough revisions were made together with all authors for significant intellectual contribution.

## Conflicts of Interest

The authors declare no conflicts of interest.

## Supporting information


Data S1.

## Data Availability

The data that support the findings of this study are available from the corresponding author upon reasonable request.
