# Supplementary material for: Carers’ Understanding of Recovery‐Oriented Practice in Mental Health Settings: A Systematic Review and Narrative Synthesis
Source: Int J Ment Health Nurs. 2025 Apr 7;34(2):e70035. doi: 10.1111/inm.70035 (PMC11976143; doi:10.1111/inm.70035)
Supplement: Supplementary file 1 — Data S1. [file INM-34-0-s001.docx]

Supplementary file-1: Concept table and search strategy

| Concept-1  (**Population**) | Concept-2  **(Exposure**) | Concept-3  (**Context**) | Concept-4  (**Concept**) |
| --- | --- | --- | --- |
| Carers | Recovery-oriented services | Mental health/illness | Perspectives |
| Carer*  Caregiver*  Famil*  Friend*  Relative*  Attendant* | Recover*  "Person-centered care"  "Psychiatric rehabilitation*"  "Mental health service*" | "Mental health"  "Mental illness*"  "Mental disorder*****"  "Mental disease"  Psychiatr*  "Depression"  "Psychotic"  "Psychosis" "Schizophrenia"  "Bi?polar disorder*" "Anxiety disorder*" "Posttraumatic stress disorder"  "Psychiatric patient*"  "Mental health Challenge*" | Understand*  Perception*  Perspective*  Explor*  Operationali*  Conceptuali*  Knowledge*  View*  Attitude*  Awareness*  Experience*  Value*  Mean* |

Search strategy- developed in Ovid database form

**P**- (Carer* OR Caregiver* OR Famil* OR Friend* OR Relative* OR Attendant*)

**E**- (Recover* OR "Person-centered care" OR "Psychiatric rehabilitation*" OR "Mental health service*")

**C**- ("Mental health" OR "Mental illness" OR "Mental disorder*****" OR "Mental disease" OR Psychiatr* OR "Depression" OR "Psychotic" OR "Psychosis" OR "Schizophrenia" OR "Bipolar disorder*" OR "Bi-polar disorder*" OR "Anxiety disorder*" OR "Posttraumatic stress disorder" OR "Psychiatric patient*" OR "Mental health challenge*")

**O-** (Understand* OR Perception* OR Perspective* OR Explor* OR Operationali* OR Conceptuali* OR Knowledge* OR View* OR Opinion* OR Attitude* OR Awareness OR Experience* OR Value* OR Mean*)

**For population concept in the research title: Not** (Child* OR Paediatric* OR Pediatric* OR Adolescen* OR Teen* )
